# Editorial: Methods in pediatric infectious diseases 2024

**DOI:** 10.3389/fped.2025.1712605

**Published:** 2025-10-23

**Authors:** Ioannis Kosmas, Gabrielle A. Mota, Zhongjie Shi

**Affiliations:** ^1^ General Hospital of Ioannina G. Hatzikosta, Ioannina, Greece; ^2^ Neurohistology Laboratory, Center for Computation, Mathematics and Cognition, Federal University of ABC, Sao Bernardo do Campo, Brazil; ^3^ Department of Pediatrics, Wayne State University, Detroit, MI, United States

**Keywords:** pediatrics, infectious diseases, method, diagnosis, treatment

**Editorial on the Research Topic**
Methods in pediatric infectious diseases 2024

As a continued Research Topic of the previous “*Methods in Pediatric Infectious Diseases 2022*” (1), this Research Topic received more attention and submissions, partially due to the influence of the global Covid-19 pandemic (2). The methods applied in understanding the immune mechanisms and clinical manifestations of pediatric infections continue to evolve, reflecting both the challenges and opportunities in advancing pediatric care. Within this topic, fourteen articles are presented that describe important and recent findings in pediatric infectious diseases.

During the COVID-19 pandemic, children were the least affected group, but those cases exhibited a wide range of clinical manifestations, ranging from asymptomatic to severe conditions. In order to better understand the impacts of SARS-CoV-2 Omicron, Sun et al. conducted an observational cohort study to describe the outcomes of severe and critically ill children infected in northeastern China and found that respiratory failure and COVID-19-associated neurological disorders were the most common complications. They pointed out that chest computed tomography (CT) score, the Pediatric Logistic Organ Dysfunction-2 (PELOD-2) score, and serum aspartate aminotransferase (AST) were important indicators of poor outcomes in children. Takane-Cabrera et al. led a similar methodology aiming to describe the characteristics and risk factors associated with disease severity across six waves of COVID-19 in children in Mexico. They found that the most affected children were the 12–17-year-old group. However, the 0–2-year-old group had higher rates of hospitalization, ICU admission, and case fatality rate (CFR), meaning that children under two years of age had the worst outcomes.

Multisystem inflammatory syndrome in children (MIS-C) is a severe complication of the COVID-19 infection. In order to understand the effectiveness of biologic and target-synthetic therapies in preventing MIS-C development, Khoury et al. conducted a retrospective cohort study based on the major Israeli health organization. Children aged 0–18 years who tested positive for COVID-19 were included, and none of the individuals who received treatments developed MIS-C. These authors suggested a possible association between biological and target-synthetic therapies and reduced risk of MIS-C following COVID-19 infection in children.

Acute bronchiolitis is the most common respiratory infection and a major cause of hospitalization in infants. Cortazzo et al. evaluated the clinical relevance of bacterial and/or viral respiratory coinfection in infants younger than three months old hospitalized with bronchiolitis in Rome. The statistical analyses revealed that there is a correlation between respiratory coinfection and a longer hospital stay and use of invasive mechanical ventilation. The same association was not seen in the viral mono-infection group. Additionally, premature infants were found to be at higher risk of respiratory coinfections compared to viral mono-infections.

Through the years, vaccination campaigns have been able to significantly decrease the incidence of many diseases, as exemplified by the Covid-19 pandemic (3). But what is the role of pediatricians when facing a vaccine-resistant family? Moorthy et al. answered this tough but important question and pointed out a possible solution. The idea was to compromise with the family to develop a vaccine schedule that was not complete, since partial immunity was better than no immunity. An adapted vaccine schedule should be proposed, and a more effective vaccine (4) would be more likely to be accepted by the family when factors such as disease severity and mortality risk, availability of treatment, vaccine risk profile, what diseases were actively spreading in the patient's community, and potential threats to public health were carefully considered (5). Likewise, Starshinova et al. presented a review on the history of the Bacillus Calmette–Guérin (BCG) vaccine and how the genetic alterations in BCG strains have evolved from the original variant. Besides the anti-tuberculosis effect, the BCG vaccine offered protection against infections involving mucous membranes showing how important it was to study the variety of protective benefits of this vaccine.

*Mycoplasma pneumoniae* (MP) is the major pathogen causing community-acquired pneumonia (CAP) in children 5 years old or older. Ding and Jiang showed in a recent review that some of the most common biomarkers in clinical practice, such as C reactive protein (CRP), procalcitonin (PCT), and serum amylase A (SAA), were good tools for clinical use because of their high sensitivity in the early diagnosis of MP pneumonia. Besides that, CRP and LDH measures were able to predict treatment courses and the patient's response. The authors concluded that the affordable and convenient blood work and cytokine-based markers, joined with these indicators, improved the accuracy of the diagnosis. Zhang et al. reported a rare case of a 9-year-old boy with refractory MP pneumonia complicated by bilateral pulmonary embolism and pulmonary infarction. Initially, the patient was treated with the conventional protocol for MP pneumonia, but after laboratory exams and chest CT, he met the diagnostic criteria for both severe MP and refractory MP pneumonia. The treatment was approached with a multidisciplinary protocol combining anti-infective agents, anti-inflammatory therapy, and adjusted anticoagulation. The patient had a rapid recovery with some residual sequelae.

Furthermore, one of the possible complications of MP pneumonia in children is plastic bronchitis (PB). Although rare, it is a severe condition that involves the formation of obstructions to the airways and can lead to respiratory failure if not treated promptly. In this retrospective cohort study, Lian et al. developed and validated a nomogram incorporating the CD4+/CD8+ ratio to predict PB in children who underwent bronchoscopy. Fever duration, presence of atelectasis, elevated D-dimer, and reduced CD4+/CD8+ ratio were identified as independent predictors of PB in MP pneumonia. Such a tool, together with other potential non-invasive imaging tools (6–8), could support bronchoscopy decision-making and optimize outcomes in pediatric cases of MP pneumonia. Another possible complication is the parapneumonic effusion (PPE), a pleural effusion caused by infectious and non-infectious disorders. Although the incidence of severe PPE has declined since the introduction of the 13-valent pneumococcal conjugate vaccines, immunological screening in children is of the most importance since early diagnosis and adequate treatment is directly connected to better outcomes. Rószai et al. investigated the immunological function of children with severe PPE during hospitalization and after full recovery through a prospective study. The duration of hospitalization was longer in the immunocompromised group when compared to the non-immunocompromised group. Within the first group, there were immunodeficiency virus infection, immunoglobulin A deficiency, mannose-binding lectin deficiency, and specific antibody deficiency.

The clinical use of biomarkers for diagnoses, especially for the early detection of infections in children, is extremely important and essential for positive prognoses. Yin et al. led a retrospective study to evaluate the most commonly used markers for neonatal late on-set sepsis (LOS). The markers analyzed included neutrophil to lymphocyte ratio (NLR), platelet to lymphocyte ratio (PLR), CPR, platelet-to-neutrophil ratio (PNR), and procalcitonin. ROC analysis showed high levels of specificity and sensitivity, pointing to the potential of NLR and PLR as reliable biomarkers for LOS diagnosis. Additionally, the combination of NLR, PLR, and CRP further improved diagnostic accuracy.

Cytomegalovirus (CMV) is a virus part of the *Herpesviridae* family and is a leading cause of congenital infections, which have a high and damaging incidence in preterm infants. Li et al. described a case report of a 2-day-old child delivered at 36 + 2 gestation weeks who had scattered bleeding spots across the body, hemorrhagic diathesis, thrombocytopenia, positive blood CMV IgM, and high levels of CMV DNA in blood and urine. The infection caused CMV retinitis, which was a common result of CMV infection in patients with immunodeficiencies. The treatment chosen was an antiviral protocol with Ganciclovir.

Pulmonary echinococcosis is a parasitic infection associated with high morbidity and mortality rates and is especially relevant in endemic regions. China accounts for more than 70% of all recorded cases worldwide. Li et al. reported the case of a 13-year-old girl who was initially misdiagnosed with pulmonary tuberculosis. After surgical intervention and histopathological examination, the diagnosis of pulmonary echinococcosis was confirmed, and the patient received Albendazole treatment and symptomatic management, which led to full recovery.

It is well known that pediatric leukemia is an important health issue in childhood. Although the survival rates of pediatric leukemia are higher than 65%, infections may increase the morbidity and mortality in children. Li et al. analyzed patients with acute lymphoblastic leukemia (ALL) and acute myeloid leukemia (AML) to determine the prognosis of different types of acute infection in pediatric leukemia patients. The authors found that the incidence of pneumonia and sepsis is significantly higher in the AML group. Besides that, younger children in both groups had more favorable prognoses than older children. Nanomedicine or other factors could be a promising way to treat those patients (9, 10).

In conclusion, pediatric infectious diseases have caused a serious global public health problem. Therefore, to understand its latest diagnosis, prognosis, and treatment methods are of the utmost importance. Factors such as efficient clinical predictors for early diagnosis and adequate treatment must be continuously studied and widely disseminated.
